# Supplementary material for: Multi-Species Host Use by the Parasitoid Fly Ormia lineifrons
Source: Insects. 2023 Sep 5;14(9):744. doi: 10.3390/insects14090744 (PMC10531574; doi:10.3390/insects14090744)
Supplement: Supplementary file 1 [file insects-14-00744-s001.zip › insects-2570243-supplementary.pdf]

Supplementary Material

Multi-species host use by the parasitoid fly Ormia lineifrons by Rogers and Beckers

Statistics

We used a linear model to compare the mass of all pupae, i.e., pupae that did and did not successfully develop into adult flies with ‘species’, parasitoid load’ and the interaction as fixed effects. We used post-hoc Tukey HSD tests to compare significant main effects. The interaction was not significant and was removed from the model. We used the software R (Version 1.4.1106) to calculate the model.

Results

Similar to the analysis of only successfully developing larvae, pupal mass differed significantly across species (ANOVA, $F_{3,80} = 7.77$, $p < 0.001$) with *N. velox* pupae being significantly lighter than those of the other hosts species (all post-hoc Tukey HSD tests: $p \leq 0.039$), whereas the mass of the other three host species did not differ (all post-hoc Tukey HSD tests: $p \geq 0.470$). Pupal mass decreased with parasitoid load (ANOVA, $F_{1,80} = 49.09$, $p < 0.0001$; Fig. S1).

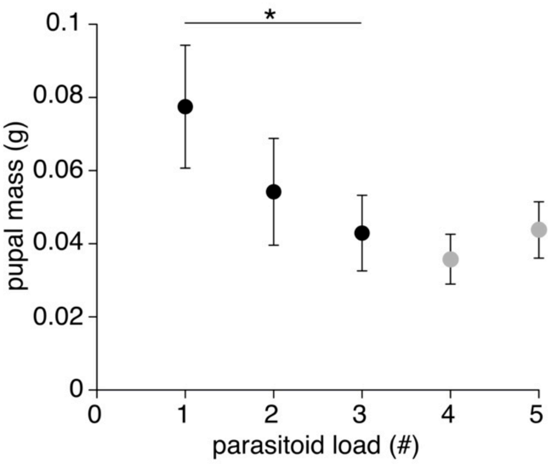

Figure S1

Average mass (± S.D.) of all pupae that emerged from *Neoconcephalus* hosts in relation to the hosts' parasitoid load (1-3 parasitoids/host; n = 16-38). Grey symbols indicate masses with low sample sizes (4-5 parasitoids/host, n = 2-4). Asterisk indicates significant difference.
